# Effectiveness of fecal-derived microbiota transfer using orally administered capsules for recurrent *Clostridium difficile* infection

**DOI:** 10.1186/s12879-015-0930-z

**Published:** 2015-04-17

**Authors:** Bruce E Hirsch, Nimit Saraiya, Kaitlin Poeth, Rebecca M Schwartz, Marcia E Epstein, Gerard Honig

**Affiliations:** The Karin and Dayton Brown, Jr. Division of Infectious Diseases, Department of Medicine, North Shore-Long Island Jewish Health System & Hofstra North Shore-LIJ School of Medicine, 400 Community Drive, 11030 Manhasset, New York USA; Department of Population Health, North Shore-Long Island Jewish Health System & Hofstra North Shore-LIJ School of Medicine, Manhasset, New York USA; Symbiotic Health Inc., New York, New York USA

**Keywords:** *Clostridium difficile*, Fecal transplantation, Fecal microbiota transfer, Capsule, Diarrhea, Microbiota, Microbiome, Fecal microbiota transplantation

## Abstract

**Background:**

*Clostridium difficile* infection (CDI), a complication of antibiotic-induced injury to the gut microbiome, is a prevalent and dangerous cause of infectious diarrhea. Antimicrobial therapy for CDI is typically effective for acute symptoms, but up to one third of patients later experience recurrent CDI. Fecal-derived microbiota transplantation (FMT) can ameliorate the underlying dysbiosis and is highly effective for recurrent CDI. Traditional methods of FMT are limited by patient discomfort, risk and inefficient procedures. Many individuals with recurrent CDI have extensive comorbidities and advanced age. Widespread use of FMT requires strategies that are non-invasive, scalable and applicable across healthcare settings.

**Methods:**

A method to facilitate microbiota transfer was developed. Fecal samples were collected and screened for potential pathogens. Bacteria were purified, concentrated, cryopreserved and formulated into multi-layered capsules. Capsules were administered to patients with recurrent CDI, who were then monitored for 90 days.

**Results:**

Thirteen women and six men with recurrent CDI were provided with microbiota transfer with orally administered capsules. The procedure was well tolerated. Thirteen individuals responded to a single course. Four patients were cured after a second course. There were 2 failures. The cumulative clinical cure rate of 89% is similar to the rates achieved with reported fecal-derived transplantation procedures.

**Conclusions:**

Recurrent CDI represents a profound dysbiosis and a debilitating chronic disease. Stable cure can be achieved by restoring the gut microbiome with an effective, well-tolerated oral capsule treatment. This strategy of microbiota transfer can be widely applied and is particularly appropriate for frail patients.

**Electronic supplementary material:**

The online version of this article (doi:10.1186/s12879-015-0930-z) contains supplementary material, which is available to authorized users.

## Background

*Clostridium difficile* infection (CDI) has been identified by the Centers for Disease Control and Prevention as one of the most prevalent hospital-acquired infections in the United States [1] and an “immediate public health threat that requires urgent and aggressive action” [2]. There are an estimated 453,000 cases of CDI per year in the US, with 29,000 associated deaths [3,4]. CDI leads to multiple morbidities [2,5] and billions of dollars in excess medical costs [6-8]. The only widely available treatments for recurrent CDI are antimicrobials directed against the pathogen. While this is generally effective for acute symptoms, up to one third of patients experience recurrent infection, and with each failure of antibiotic treatment, chances of later successful treatment diminish [3,7,9-13]. Recurrent CDI has been defined as the recurrence or continuation of diarrhea symptoms, with a positive diagnostic test for *Clostridium difficile* in the stool, after the completion of therapy for initial CDI episode [14]. Patients with CDI, especially the recurrent form, experience severe morbidity, impaired functioning and risk of clinical decompensation, and pose prolonged infectious risk to others. Hypervirulent strains of *Clostridium difficile* have emerged since 2000 and are associated with particularly high risk of recurrent CDI [15].

CDI is a disorder of the gastrointestinal (GI) microbiota. The human-associated microbiota is a dense and diverse population of symbiotic bacteria which use the human body (particularly the colon) as habitat [16]. The microbiota has co-evolved with humans and plays essential roles in health and disease. One of its most crucial roles is colonization resistance, or the prevention of overgrowth by pathogenic bacteria via direct competition and additional mechanisms [17-19]. When the microbiota is injured, most often following oral antibiotic administration, colonization resistance is impaired, allowing pathogens such as *Clostridium difficile* to proliferate and dominate the gut ecosystem [17]. Antibiotics targeted against CDI may eradicate the active infection, but do not redress (and may even exacerbate) long-lasting dysbiosis of the microbiota, the major risk factor for relapse [20].

The role of the microbiota in antibiotic-induced diarrhea was recognized long before the identification of specific pathogens responsible for this condition. This understanding has stimulated significant interest in fecal-derived microbiota transfer (FMT): the transplantation of fecal-derived bacteria from a healthy individual into the GI tract of the affected patient [21-25]. This strategy, which utilizes the complex fecal microbiota of a healthy donor to reconstitute the normal microbiota, is an effective and appropriate treatment for recurrent CDI or pseudomembranous colitis, as supported by many case series reports [11,22,25-51] and a randomized trial [52]. FMT can be administered by various modalities including instillation of donor fecal content by colonoscopic or nasogastric intubation [28]. In the present work, we describe the use of orally administered capsules to deliver cryopreserved fecal-derived bacteria for the treatment of recurrent CDI.

## Methods

### Inclusion criteria

FMT by capsule for the treatment of recurrent CDI is offered as a standard treatment by the North Shore LIJ Division of Infectious Diseases. The present work represents a retrospective chart review of all such cases encountered between April 2013 and February 2014. These dates were determined by the North Shore LIJ Institutional Review Board, and did not represent start or stop of a clinical trial. Eligible candidates for the procedure are individuals with two or more prolonged, severe and/or recurrent episodes of CDI after therapy with metronidazole, vancomycin, and/or fidaxomicin. Subjects were adults over 18 years of age who were receiving antibiotic treatment for CDI at the time of evaluation and were capable of swallowing capsules. Exclusion criteria included gastroparesis, neutropenia, ileus, and hemodynamic instability.

### Ethics & compliance

All work was evaluated and approved by the Institutional Review Board of the North Shore-Long Island Jewish Health System. All procedures were performed under and compliant with the current policy of the Food and Drug Administration on the use of FMT for recurrent CDI [53]. An Investigational New Drug application was submitted to the Food and Drug Administration prior to the issuance of the current guidance, but later withdrawn at the FDA’s recommendation.

### Fecal matter donation

Donors were three healthy volunteers unrelated to the recipients. Individuals in good health who had not received antibiotics for at least 6 months prior to the procedure were considered as candidates for stool donation. Individuals with a history of irritable bowel disease, inflammatory bowel disease, body mass index over 25, diabetes mellitus or psychiatric illness were excluded. Extensive screening for potential pathogens in the blood and feces was performed using standard clinical diagnostic protocols at the North Shore-LIJ Core Laboratory. Testing was done on-site except where noted. Following a standard small-volume venous blood draw, the following serologic tests were performed by chemiluminescent microparticle immunoassay: combined HIV-1 and HIV-2 Ab/Ag; Hepatitis A (total anti-hepatitis A virus IgG/IgM); Hepatitis B (HbsAg and anti-Hbs); and Hepatitis C (anti-Hepatitis C Virus). Treponema pallidum particle agglutination test was performed for syphilis. Enzyme immunoassay for Human T-Lymphotrophic Viruses types I and II was performed at Mayo Medical Laboratories (Rochester, NY). At the time of stool donation, the sample was evaluated as follows. PCR for the toxin B gene of *Clostridium difficile* was performed using the BD GeneOhm Cdiff Assay according to the manufacturer’s instructions. Stool was plated by a clinical microbiologist using the following media: 5% sheep blood agar; MacConkey agar; MacConkey agar with sorbitol; and Hektoen enteric agar plates were incubated aerobically at 35°C. CIN *Yersina* selective agar plates were incubated at room temperature. Campylobacter agar plates were incubated at 42°C anaerobically using the gas-pack method. Plates were examined by an experienced observer for *Salmonella, Shigella, Campylobacter, E. coli O157, Yersinia, Vibrio, Aeromonas and Plesiomonas* colonies. For ova and parasite testing, an additional stool sample was collected into fixative and evaluated by microscopy. A donor sample was used for FMT if and only if a complete set of negative screening results had been received for the donor, not more than 30 days prior to the date of sample collection. No instances of disqualifying test results were obtained from such screening. Between screening and donation, donors were instructed to abstain from antibiotics; bismuth preparations; undercooked meat, poultry, seafood or eggs; travel outside of the United States; and any known infectious contacts. Donors were queried for any intercurrent illness at the time of donation. For fecal sample collection, donors were received in an outpatient clinic by a healthcare provider who performed screening and provided guidance regarding sample collection. Sample collection was performed by the donor at the clinic using sterile transfer materials and appropriate personal protective equipment. The sample was then remitted to the healthcare provider, who then submitted it to a laboratory technician for processing.

### Capsule formulation

Processing was performed with strict environmental control not later than 6 hours following defecation. All vessels used for sample handling were sterile, sealed single-use plastic (Nalgene), with the exception of a homogenizer (Black & Decker) which was disinfected and washed with 10% bleach, detergent and water prior to use. All reagents were sterile USP grade. Wet weight of stool was recorded; stool was then homogenized briefly in 350 mL 0.9% NaCl in water and aliquoted into sterile disposable conical centrifuge tubes. Samples were then centrifuged at 200 g for 10 minutes. Upon removal from centrifuge, a pellet of insoluble material formed. Supernatant was then decanted into new centrifuge tubes and centrifuged at 4600 g for 15 minutes. Two layers of pellet typically formed: a lower pellet of insoluble material of variable size, and a larger, dense bacterial pellet. The supernatant was removed and the upper pellets were re-suspended in 0.9% sodium chloride with a final concentration of 15% glycerol (v/v). Concentration was calculated as the wet weight of input material over the final slurry volume; typical concentration for preparations used was 0.5 g/mL. The resulting FMT slurry was used to fill 5 mL or 10 mL syringes and immediately frozen over dry ice and stored at −80°C. Duration of storage at −80°C was 1–3 weeks. Prior to use, syringes were transferred to −20°C storage and used within 6 weeks. Immediately prior to FMT, aliquots were thawed. Aliquots of 0.4 mL of FMT slurry were dispensed into Size 1 acid-resistant hypromellose capsules (DRCaps, manufactured by Professional Compounding Centers of America, Houston, TX), subsequently placed within Size 0 acid-resistant hypromellose capsules and then nested within Size 00 gelatin Caps (Capsuline, Pompano Beach, FL). Capsules were administered immediately upon filling and capping.

### FMT procedure

Informed consent was obtained from each patient. Antimicrobial treatment for CDI was discontinued the day prior to FMT. A proton pump inhibitor was given on the evening and the morning prior to the procedure. Patients were instructed to eat lightly on the morning of the procedure. Under direct supervision in an out-patient office setting, the patients ingested 6–22 capsules (average of 10 ± 3 standard deviation) and were monitored for and immediate adverse effects. Each patient was offered 8–12 capsules. One patient (see Subject S, Additional file 1: Table S2) developed anxiety and declined additional capsules after ingesting only 6. This patient’s initial course was the only instance in which the number of capsules was limited due to the patient’s request. This same individual failed treatment despite later being provided with an augmented dose of 22 capsules at his family member’s request. He had no difficulty ingesting the capsules on subsequent courses. Patients were instructed to not eat and to remain upright for 1 hour after ingesting capsules. The procedures were performed between April 2013 and February 2014.

### Estimation of dose

The mean mass of initial fecal matter used to produce a successful FMT dose was 2.3 g. Although we did not quantitatively assess the density and viability of bacteria delivered, we can estimate the bacterial load transferred based on results from a flow cytometry study of bacterial population in fecal matter from healthy individuals [54]. According to that work, feces contains, on average, 8.6×10^10^ bacterial cells per gram of wet weight; vital staining indicated that 49% had an intact cell membrane (conservative marker of viability) for an estimated 4.2×10^10^ intact bacteria per gram input material. Thus we estimate that a typical FMT dose contained 9.7×10^10^ viable bacteria at the time of initial production. An estimate of the input material corresponding to each treatment instance is provided in a table (see Additional file 1: Table S2).

### Adjunctive procedures

Patients were encouraged to consume 4 ounces of Kefir fermented milk product twice daily and were given a list of “pre-biotic” nutrients to consume for at least 3 days after FMT. In the circumstance of persistent or recurring CDI, patients were instructed to immediately resume CDI antimicrobial therapy and report their clinical status.

### Outcomes

Patients were followed by phone interview within 2 days, within 3 weeks and after 90 days to assess their response to the procedure. They were instructed to promptly report clinical changes and were specifically evaluated for the recurrence of symptoms associated with their previous episodes of CDI-associated diarrhea. Diarrhea was defined as ≥ 3 unformed stools within 24 hours. They were simultaneously assessed for adverse effects. Patients with recurrent CDI were re-treated with anti-microbial therapy and subsequently offered repeat FMT. When performed, sequential FMT treatments were administered approximately six weeks after the previous FMT. After each instance of FMT, follow-up was extended for an additional 90 days. The primary end point was resolution of CDI associated diarrhea without relapse after 90 days. Recurrence was defined as diarrhea with positive stool testing for *Clostridium difficile* toxin B by PCR. Diarrhea explained by other causes, responding to etiology-specific therapy, with negative stool tests for *Clostridium difficile* toxin B, were not considered as FMT failures. Cases were tabulated and mean values and proportions were calculated.

## Results

A total of 19 patients with recurrent CDI were identified as candidates for FMT. Patient demographics, medical history and FMT treatment details are indicated (see Additional file 1: Table S2). Patient ages ranged from 26–92 years, with a mean of 61 years. There were 13 females and 6 males. The patients had an average of 4 CDI recurrences (range, 2–8). Co-morbid conditions were common (average of 2.5 per patient) and are indicated in Table 1. Only one patient had no other chronic condition. FMT was administered in the outpatient setting in all instances.

**Table 1:** **Comorbidities in the study population**

**Comorbidities**	**Number of patients (of 19)**
Cardiovascular	14
Atrial fibrillation	3
Hypertension	8
Cardiomyopathy	1
Coronary artery disease	2
Endocrine	8
Diabetes	2
Polycystic ovary syndrome	1
Hypothyroidism	2
Thyroid nodule	1
Obesity	2
Gastrointestinal	6
Chronic pancreatitis	2
Irritable bowel syndrome	3
Diverticulitis	1
Hematological malignancy	2
Lymphoma	1
Acute myeloid leukemia	1
Solid Malignancies (Renal carcinoma)	1
Immunological (psoriasis)	1
Neurological	8
Dementia	3
Parkinson’s	1
Stroke	1
Chronic pain	3
Psychiatric	3
Anxiety	1
Depression	2
Pulmonary (asthma)	2
Chronic renal insufficiency	1

The primary endpoint was lasting resolution of CDI diarrhea, assessed 90 days after the last FMT. Thirteen patients (68%) had resolution after a single instance of FMT treatment. Of 6 patients that did not respond to the initial treatment, 4 went on to have resolution after one subsequent FMT treatment, for a cumulative cure rate of 89%. Repeat FMT procedures were performed approximately six weeks after the initial FMT. There was only one patient (Subject S, Additional file 1: Table S2) who had more than 2 treatments; he received 4 treatments without long term resolution of diarrhea. In successful courses, clinical improvement was noted within three days of FMT; in most cases, symptoms gradually improved over the course of two weeks. The FMT procedure was well tolerated. There was no distaste, respiratory distress nor immediate discomfort with ingestion of capsules. There were no infectious complications. Abdominal pain was reported post-procedure by five subjects. The pain was mild and transient in four cases. There was one instance of moderate to severe epigastric distress which required narcotic analgesics. The corresponding patient (Subject G, Additional file 1: Table S2) was a 44-year old woman with chronic abdominal pain and irritable bowel syndrome (IBS) in addition to recurrent CDI. She was hospitalized five days after FMT. Abdominal CT scan was negative, repeat testing for *Clostridium difficile* toxin B by PCR was negative and pain improved after 3 days. The clinical presentation was inconsistent with viral gastroenteritis. The pain was similar to her previous pain episodes. Patients with IBS did not have improvement in IBS symptoms after FMT.

There were two FMT failures. A 76-year-old man (Subject O, Additional file 1: Table S2), with 3 CDI recurrences, diabetes mellitus, hypertension, coronary artery disease and renal cell carcinoma, experienced soft stools 11 days after FMT and was provided a course of oral vancomycin by his primary care physician. He remained free of CDI after an additional 90 days. The second case (Subject S, Additional file 1: Table S2) was an 84 year old man with dementia, coronary artery disease, congestive heart failure, arthritis and non-Hodgkins’ lymphoma who had recurrent symptoms consistent with CDI within 2–4 weeks of each of 4 FMT attempts. He required hospitalization for diarrhea, dehydration and acute kidney injury. The diarrhea resolved with oral vancomycin and rifaximin. He developed health-care acquired pneumonia which was treated with systemic antimicrobial therapy and oral vancomycin was continued. He did not develop recurrent diarrhea. He expired from respiratory failure. His death was considered unrelated to the FMT procedure.

## Discussion

Conventional treatment options for CDI have significant limitations and are mostly ineffective for the recurrent form of the infection. Metronidazole and oral vancomycin are standard therapies. Fidaxomicin has been the only additional pharmaceutical to be approved by the Food and Drug Administration for CDI over the past 25 years; although its use is associated with lower rates of recurrence [6], concerns regarding its cost-effectiveness have limited its adoption [6,55-57]. Although most patients with CDI experience acute relief with antibiotic therapy, diarrhea recurs in up to 35% of patients after an initial episode [58] and up to 60% of patients after a third episode [11]. Additional strategies include oral vancomycin with an extended taper; rifaximin; intravenous tigecycline; toxin binding resins, monoclonal antibodies and various FMT modalities [29,42,45,59].

The gut lumen comprises an exceptionally dense and complex microbial ecosystem [60,61].The gut microbiome participates in diverse aspects of human biology including nutrient absorption, gastrointestinal function, colonization resistance against potential pathogens and immunomodulation [62]. Vulnerability to CDI is related to the microbial composition of the gut. Comparative metagenomic analysis has demonstrated alterations in microbiota at the phylum level. In one study, when compared with controls, patients with *Clostridium difficile*-associated disease had increases in Firmicutes, Proteobacteria and Actinobacteria with decreases in Bacteroidetes and in overall diversity of the gut microbiome [63]. Antibiotic exposure is the most common risk factor for CDI. Other risk factors include proton pump inhibitors, advanced age, gastrointestinal surgery and serious underlying illness or comorbidities [64]. Antibiotic administration is associated with long term changes in gut microbiota. For example, significant alterations in gut microbiome were found to persist two years after a 7-day course of clindamycin [65].

FMT has been shown to be effective using multiple methods of administration. Introduction of fecal-derived bacteria into the gut can be accomplished in numerous ways [37,66]. Successful FMT is associated with marked change of gut microbiota with enhanced diversity and increase in relative abundance of members of the genus Bacteroides [67]. An early case report described dramatic clinical improvement of acute severe CDI associated with FMT performed by enema [68]. A high success rate for recurrent CDI was documented with donor stool administration via nasogastric tube [69]. Multiple reports document excellent results with colonoscopic instillation of fecal-derived bacteria into terminal ileum and cecum [70-72]. A recently published randomized clinical trial evaluated nasoduodenal infusion of donor feces versus oral vancomycin; this trial was terminated prematurely as the remarkably high rate of success of FMT raised ethical concerns about continued administration of the antibiotic control treatment [52].

At present, the mechanism whereby FMT leads to resolution of recurrent CDI is unclear. It is thought that a sufficiently complex bolus of bacteria reverses the suppressive effect of the pathogen on the endogenous microbiome, allowing the re-establishment of colonization resistance [73]. Restoration of the gut microbiome may inhibit sporulation, germination and/or vegetative growth *of Clostridium difficile*. A few patients in this study had continuous diarrhea despite ongoing antimicrobial therapy. In these cases, symptom relief could be observed as rapidly as 48 hours post FMT, suggesting that even the small number of bacteria present in the FMT dose can act, via an unknown mechanism, to alleviate symptoms prior to full reconstitution of a diverse endogenous microbiome. Correction of metabolic dysfunction has been suggested to play an important role [67]. A recent study associated the presence of *Clostridium scindens* with resistance to CDI and correlated the ability of this organism to generate secondary bile acids, inhibiting the growth of *Clostridum difficile* [74].

In the present work, we integrated previous approaches to develop an innovative treatment protocol for FMT. After homogenization with normal saline, low-speed centrifugation was performed to remove particulate matter and then high speed centrifugation to concentrate bacteria and reduce the final volume. Resuspension of the bacteria in 15% glycerol for cryopreservation has been previously validated to retain clinical activity [25,75]. Encapsulation of stool material has been presented in abstract form (Louie TJ et al., ‘Microbiome profiles of recipients and donors post fecal transplantation for multiple recurrent CDI infection,’ presented at 3rd International *Clostridium difficile* Symposium, Slovenia) and has been described in a publication released while this manuscript was under review [76] (see discussion below). With our protocol, the concentration of fecal-derived bacteria necessitated fewer capsules which may enhance ease of administration. Cryopreservation rendered the process safer and practical by obviating the need for precise coordination of donor sample collection and by facilitating extensive pathogen screening of each donor sample. Our use of nested delayed-release capsules within a smooth opaque gelatin capsule was aesthetically acceptable and easily managed by our patient population while promoting effective delivery to the distal GI tract.

We unexpectedly observed that the minimum effective dose of bacteria for effective FMT was much lower than the typical doses reported for the treatment of recurrent CDI. Using the processing and encapsulation methodology applied here, rapid clinical efficacy was observed in 70% of patients with a dose derived from less than 3 g of input material, with an estimated 9.7×10^10^ viable bacteria per course. Typical FMT protocols described utilize 50–60 g for a single dose administered by colonoscopy, with an entire fresh stool sample processed in order to generate a single dose [28]. Importantly, however, one-time FMT by colonoscopy has been reported to be more effective than our minimum dose [40], whereas our treatment protocol with 1–2 capsule doses was, cumulatively, as effective as colonoscopic FMT, suggesting that our initial dose may be lower than optimal. We are evaluating alternative dosing schedules and delivery methods accordingly. The more efficient use of donor material has major advantages. It is possible to generate a large number of doses from a single instance of processing, dramatically reducing costs associated with pathogen screening. FMT material retained therapeutic efficacy following 6 weeks of −20°C storage. In addition, extensive pre-treatment measures such as bowel lavage were not required for clinical efficacy. Taken together, these advantages could allow this treatment modality to be applied across many healthcare settings such as long-term care facilities.

Three different donors were used to generate capsules used in this case series. Material derived from all three donors was clinically effective. No significant differences in time to resolution of symptoms or efficacy among the different donors were noted in this small sample. Future studies with larger sample sizes would be required to identify a dose–response relationship. For future investigation, we plan to apply metagenomic analysis in an attempt to better understand the recovery from marked dysbiosis in recurrent CDI patients. An additional area for future work includes more comprehensive screening for potential asymptomatic low-level carriage of pathogens in donors. Clinically validated tests for enteric pathogens are generally designed to detect active infection. We are currently developing approaches to detect a wider range of bacterial and viral pathogens with high sensitivity to improve the donor screening process. Patients received a range of dosages. The high success rate, small sample size and range of dosages precluded our ability to determine a dose–response relationship.

Adjunctive measures were utilized to augment the effectiveness of FMT. Acute administration of a proton pump inhibitor was used in an attempt to reduce the potential loss of bacterial viability from stomach acid. The acid-resistant nature of the capsules may render this measure unnecessary; we are performing *in vitro* viability assessment to clarify this. Kefir fermented milk product has been described as beneficial in conjunction with antibiotic therapy for CDI [77]. In that study a staggered and tapered antibiotic withdrawal was combined with regular ingestion of kefir liquid was effective in preventing diarrhea in 21/25 cases for 9 months following these treatment. The use of short term kefir product may have augmented efficacy in our study. However, a multicenter randomized trial recently reported a lack of efficacy of a widely used probiotic regimen for the prevention or treatment of CDI [78]. Furthermore, a recent study of encapsulated FMT for CDI demonstrated nearly identical clinical results without kefir, suggesting that in our study, FMT was the primary factor in clinical response [76]. Importantly, the recommended use of kefir requires frequent and long-term consumption, unlike the one- week intervention prescribed in our protocol.

The efficacy of FMT has implications for infection control and public health. The patients in this series experienced prolonged diarrhea, in one case lasting over one year. *Clostridium difficile* spores are hardy and can remain viable in the environment for 5 months [79]. In an animal model of recurrent CDI, antibiotic treatment was shown to cause a ‘supershedder’ state whereby an asymptomatic carrier sheds large numbers of *Clostridium difficile* spores; this supershedder state was suppressed by FMT in the animal model [80]. Earlier utilization of curative treatment for CDI is essential to enhance the safety of the hospital environment and to reduce the increasing frequency of community acquired infection [81].

Reimbursement for FMT has been a major limiting factor in its widespread use for recurrent CDI. This treatment is recognized by the Center for Medicare & Medicaid Services, which has assigned a specific billing code; however, the reimbursement provided is relatively low given the expense, labor, space and equipment required. The major factors in the expense associated with FMT are clinician time and pathogen screening. Coordination of donor screening, sample processing and FMT is labor-intensive, especially using traditional FMT approaches. Donor recruitment and screening for pathogens (which can cost over $1000) are generally not reimbursed. Our approach greatly improves the efficiency of this process, as many doses can be produced and banked following one instance of screening. Another limiting factor for efficient capsule production is the encapsulation process itself, which at present (like traditional FMT) requires significant hands-on clinician time immediately prior to administration. We are developing methods to improve capsule stability in order to further increase the convenience and cost-effectiveness of this treatment modality. Despite these issues, FMT for recurrent CDI is highly cost-effective, as determined in a recently published analysis [57]. In that study, even with a cost estimate of over $3,000, colonoscopic FMT was found to be the most cost effective treatment for first CDI recurrence when compared with metronidazole, vancomycin or fidaxomicin. FMT utilizing the currently described capsule method represents an efficient and non-invasive strategy which preserves clinical, epidemiologic and economic advantages of the colonoscopic approach.

Limitations of the present work include small sample size and an incomplete understanding of dose–response characteristics. However, our results are nearly identical to a recent publication describing the use of frozen fecal material administered in oral capsules [76]. That study similarly followed 20 patients with at least 3 episodes of mild to moderate CDI who had failed a 6- to 8-week taper with vancomycin or had at least 2 episodes of severe CDI requiring hospitalization. Patients were given 15 FMT capsules over 2 consecutive days and were subsequently followed for symptom resolution and adverse events for up to 6 months. Donors underwent similar screening as the present work and capsules were created and stored in a similar fashion. If patients did not achieve improvement in their diarrhea after 72 hours, they were retested and offered re-treatment if still positive. In that study, there was 70% resolution of diarrhea after a single FMT treatment (14 out of 20 patients) and an overall 90% rate of resolution (18 out of 20). The highly similar response rates observed validate the respective studies. Our study has certain additional limitations including single arm design and absence of gut microbiota evaluation. While we had follow-up on all cases, we relied on patient and family report of clinical status, reasoning that criteria for recurrence of CDI are very clear to those so afflicted.

## Conclusions

Our data suggest that FMT utilizing concentrated cryopreserved fecal-derived bacteria, administered via orally ingested capsules, is an effective, safe and well-tolerated therapy for recurrent CDI. The ease of administration facilitates multiple courses of FMT if required. We are able to demonstrate an overall success rate of 89%, with 68% experiencing cure from a single FMT. This efficacy is comparable to that of more invasive FMT modalities. Advantages of this approach include high efficacy, lack of significant adverse effects, ease of administration, patient comfort, non-invasiveness, ability to safely provide therapy to those with extensive co-morbidities and advanced age, and cost-effectiveness. This approach represents a powerful, patient-friendly and cost-efficient option for the treatment of recurrent and persistent CDI.

## Additional file

Additional file 1: Table S2. CDI history, risk factors, treatments and outcomes for patients receiving FMT. *This patient had continuous CDI diarrhea for one year despite antimicrobial treatment. **Initial improvement in symptoms was observed within 3 days; symptom improvement continued over the course of 2 weeks.

## References

[1] Magill SS, Edwards JR, Bamberg W, Beldavs ZG, Dumyati G, Kainer MA (2014). Multistate point-prevalence survey of health care–associated infections. N Engl J Med.

[2] Antibiotic resistance theats in the United States. Centers for Disease Control and Prevention; 2013, http://www.cdc.gov/drugresistance/threat-report-2013.

[3] Lucado J, Gould C, Elixhauser A (2012). Clostridium difficile infections (CDI) in hospital stays, 2009: statistical brief #124.

[4] Lessa FC, Mu Y, Bamberg WM, Beldavs ZG, Dumyati GK, Dunn JR (2015). Burden of Clostridium difficile infection in the united states. N Engl J Med.

[5] Lipp MJ, Nero DC, Callahan MA (2012). Impact of hospital-acquired Clostridium difficile. J Gastroenterol Hepatol.

[6] Kyne L, Hamel MB, Polavaram R, Kelly CP (2002). Health care costs and mortality associated with nosocomial diarrhea Due to Clostridium difficile. Clin Infect Dis.

[7] Elixhauser A, Steiner C, Gould C (2006). Readmissions following Hospitalizations with Clostridium difficile Infections, 2009: Statistical Brief #145.

[8] Dubberke ER, Olsen MA (2012). Burden of Clostridium difficile on the healthcare system. Clin Infect Dis.

[9] Kelly CP (2012). Can we identify patients at high risk of recurrent Clostridium difficile infection?. Clin Microbiol Infect.

[10] Khanna S, Pardi DS (2010). The growing incidence and severity of Clostridium difficile infection in inpatient and outpatient settings. Expert Rev Gastroenterol Hepatol.

[11] Bakken JS (2009). Fecal bacteriotherapy for recurrent Clostridium difficile infection. Anal Chem.

[12] Pépin J, Alary M-E, Valiquette L, Raiche E, Ruel J, Fulop K (2005). Increasing risk of relapse after treatment of Clostridium difficile colitis in Quebec, Canada. Clin Infect Dis.

[13] Figueroa I, Johnson S, Sambol SP, Goldstein EJC, Citron DM, Gerding DN (2012). Relapse versus reinfection: recurrent Clostridium difficile infection following treatment with fidaxomicin or vancomycin. Clin Infect Dis.

[14] Surawicz CM (2004). Treatment of recurrent Clostridium difficile-associated disease. Nat Clin Pract Gastroenterol Hepatol.

[15] Petrella LA, Sambol SP, Cheknis A, Nagaro K, Kean Y, Sears PS (2012). Decreased cure and increased recurrence rates for Clostridium difficile infection caused by the epidemic C. Difficile BI strain. Clin Infect Dis.

[16] Blaser M, Bork P, Fraser C, Knight R, Wang J (2013). The microbiome explored: recent insights and future challenges. Nat Rev Microbiol.

[17] Buffie CG, Pamer EG (2013). Microbiota-mediated colonization resistance against intestinal pathogens. Nat Rev Immunol.

[18] Tosh PK, McDonald LC (2011). Infection control in the multidrug-resistant era: tending the human microbiome. Clin Infect Dis.

[19] Abt MC, Pamer EG (2014). Commensal bacteria mediated defenses against pathogens. Curr Opin Immunol.

[20] Louie TJ, Cannon K, Byrne B, Emery J, Ward L, Eyben M (2012). Fidaxomicin preserves the intestinal microbiome during and after treatment of Clostridium difficile infection (CDI) and reduces both toxin reexpression and recurrence of CDI. Clin Infect Dis.

[21] Zhang F, Luo W, Shi Y, Fan Z, Ji G (2012). Should we standardize the 1,700-year-Old fecal microbiota transplantation?. Am J Gastroenterol.

[22] Eiseman B, Silen W, Bascom GS, Kauvar AJ (1958). Fecal enema as an adjunct in the treatment of pseudomembranous enterocolitis. Surgery.

[23] van der Waaij D, Vossen JM, Altes CK, Hartgrink C (1977). Reconventionalization following antibiotic decontamination in man and animals. Am J Clin Nutr.

[24] Heidt PJ, van der Waaij D, Vossen JM, Hendriks WD (1983). The use of a human donor flora for recontamination following antibiotic decontamination. Prog Food Nutr Sci.

[25] Youngster I, Sauk J, Pindar C, Wilson RG, Kaplan JL, Smith MB (2014). Fecal microbiota transplant for relapsing Clostridium difficile infection using a frozen inoculum from unrelated donors: a randomized, open-label, controlled pilot study. Clin Infect Dis.

[26] Vaishnavi C (2014). Fecal microbiota transplantation for management of Clostridium difficile infection. Indian J Gastroenterol.

[27] Austin M, Mellow M, Tierney WM (2014). Fecal microbiota transplantation in the treatment of Clostridium difficile infections. Am J Med.

[28] Brandt LJ, Aroniadis OC (2013). An overview of fecal microbiota transplantation: techniques, indications, and outcomes. Gastrointestinal endoscopy.

[29] Bakken JS, Polgreen PM, Beekmann SE, Riedo FX, Streit JA (2013). Treatment approaches including fecal microbiota transplantation for recurrent Clostridium difficile infection (RCDI) among infectious disease physicians. Anal Chem.

[30] Borody TJ, Brandt LJ, Paramsothy S, Agrawal G (2013). Fecal microbiota transplantation: a new standard treatment option for Clostridium difficile infection. Expert Rev Anti Infect Ther.

[31] Brandt LJ, Aroniadis OC, Mellow M, Kanatzar A, Kelly C, Park T (2012). Long-term follow-up of colonoscopic fecal microbiota transplant for recurrent Clostridium difficile infection. Am J Gastroenterol.

[32] Burke KE, Lamont JT (2013). Fecal transplantation for recurrent Clostridium difficile infection in older adults: a review. J Am Geriatr Soc.

[33] de Vos WM (2013). Fame and future of faecal transplantations – developing next-generation therapies with synthetic microbiomes. Microbial Biotechnology.

[34] Di Bella S, Drapeau C, Garcia-Almodovar E, Petrosillo N (2013). Fecal microbiota transplantation: the state of the art. Infect Dis Rep.

[35] Fuentes S, van Nood E, Tims S, Heikamp-de Jong I, ter Braak CJF, Keller JJ (2014). Reset of a critically disturbed microbial ecosystem: faecal transplant in recurrent Clostridium difficile infection. ISME J.

[36] Gens KD, Elshaboury RH, Holt JS (2013). Fecal microbiota transplantation and emerging treatments for Clostridium difficile infection. J Pharm Pract.

[37] Gough E, Shaikh H, Manges AR (2011). Systematic review of intestinal microbiota transplantation (fecal bacteriotherapy) for recurrent Clostridium difficile infection. Clin Infect Dis.

[38] Högenauer C, Kump PK, Krause R (2014). Tempered enthusiasm for fecal transplantation. Clin Infect Dis.

[39] Jorup-Ronstrom C, Hakanson A, Sandell S, Edvinsson O, Midtvedt T, Persson AK (2012). Fecal transplant against relapsing Clostridium difficile-associated diarrhea in 32 patients. Scand J Gastroenterol.

[40] Kelly CP (2013). Fecal microbiota transplantation — an old therapy comes of age. N Engl J Med.

[41] Khoruts A, Sadowsky MJ (2011). Therapeutic transplantation of the distal gut microbiota. Mucosal Immunol.

[42] Moore T, Rodriguez A, Bakken JS (2014). Fecal microbiota transplantation: a practical update for the infectious disease specialist. Clin Infect Dis.

[43] Pamer EG (2014). Fecal microbiota transplantation: effectiveness, complexities, and lingering concerns. Mucosal Immunol.

[44] Rao KMD, Young VBMDP, Aronoff DMMD (2014). Commentary: fecal microbiota therapy: ready for prime time?. Infect Control Hosp Epidemiol.

[45] Ritter AS, Petri WAJ (2013). New developments in chemotherapeutic options for Clostridium difficile colitis. Curr Opin Infect Dis.

[46] Rubin TA, Gessert CE, Aas J, Bakken JS (2013). Fecal microbiome transplantation for recurrent Clostridium difficile infection: report on a case series. Anal Chem.

[47] Russell GH, Kaplan JL, Youngster I, Baril-Dore M, Schindelar L, Hohmann E (2014). Fecal transplant for recurrent Clostridium difficile infection in children with and without inflammatory bowel disease. J Pediatr Gastroenterol Nutr.

[48] Senior K (2013). Faecal transplantation for recurrent C difficile diarrhoea. Lancet Infect Dis.

[49] Tarr P (2013). Fecal transplants for Clostridium difficile: Microbial Messiah or medical misadventure. Public Workshop: Fecal Microbiota for Transplantation: May 2013 2013; National Institutes of Health: Food & Drug Administration.

[50] Taur Y, Pamer EG (2014). Harnessing microbiota to kill a pathogen: fixing the microbiota to treat Clostridium difficile infections. Nat Med.

[51] van Nood E, Speelman P, Nieuwdorp M, Keller J (2014). Fecal microbiota transplantation: facts and controversies. Curr Opin Gastroenterol.

[52] van Nood E, Vrieze A, Nieuwdorp M, Fuentes S, Zoetendal EG, de Vos WM (2013). Duodenal infusion of donor feces for recurrent Clostridium difficile. N Engl J Med.

[53] Guidance for Industry: Enforcement Policy Regarding Investigational New Drug Requirements for Use of Fecal Microbiota for Transplantation to Treat Clostridium difficile Infection Not Responsive to Standard Therapies. Food and Drug Administration; 2013, http://www.fda.gov/BiologicsBloodVaccines/GuidanceComplianceRegulatoryInformation/Guidances/Vaccines/ucm361379.htm.

[54] Ben-Amor K, Heilig H, Smidt H, Vaughan EE, Abee T, de Vos WM (2005). Genetic diversity of viable, injured, and dead fecal bacteria assessed by fluorescence-activated cell sorting and 16S rRNA gene analysis. Appl Environ Microbiol.

[55] Bartsch SM, Umscheid CA, Fishman N, Lee BY (2013). Is Fidaxomicin worth the cost? An economic analysis. Clin Infect Dis.

[56] Juang P, Hardesty JS (2013). Role of fidaxomicin for the treatment of Clostridium difficile infection. J Pharm Pract.

[57] Konijeti GG, Sauk J, Shrime MG, Gupta M, Ananthakrishnan AN (2014). Cost-effectiveness of competing strategies for management of recurrent Clostridium difficile infection: a decision analysis. Clin Infect Dis.

[58] Khanna S, Pardi DS (2012). Clostridium difficile infection: new insights into management. Mayo Clin Proc.

[59] Lowy I, Molrine DC, Leav BA, Blair BM, Baxter R, Gerding DN (2010). Treatment with monoclonal antibodies against Clostridium difficile toxins. N Engl J Med.

[60] Lozupone CA, Stombaugh JI, Gordon JI, Jansson JK, Knight R (2012). Diversity, stability and resilience of the human gut microbiota. Nature.

[61] Liévin-Le Moal V, Servin AL (2006). The front line of enteric host defense against unwelcome intrusion of harmful microorganisms: mucins, antimicrobial peptides, and microbiota. Clin Microbiol Rev.

[62] Clemente Jose C, Ursell Luke K, Parfrey Laura W, Knight R (2012). The impact of the Gut microbiota on human health: an integrative view. Cell.

[63] Vincent C, Stephens D, Loo V, Edens T, Behr M, Dewar K (2013). Reductions in intestinal clostridiales precede the development of nosocomial Clostridium difficile infection. Microbiology.

[64] Hookman P, Barkin JS (2009). Clostridium difficile associated infection, diarrhea and colitis. World J Gastroenterol.

[65] Jernberg C, Lofmark S, Edlund C, Jansson JK (2007). Long-term ecological impacts of antibiotic administration on the human intestinal microbiota. ISME J.

[66] Agito MD, Atreja A, Rizk MK (2013). Fecal microbiota transplantation for recurrent C difficile infection: ready for prime time?. Cleve Clin J Med.

[67] Weingarden AR, Chen C, Bobr A, Yao D, Lu Y, Nelson VM (2013). Microbiota transplantation restores normal fecal bile acid composition in recurrent Clostridium difficile infection. Am J Physiol Gastrointest Liver Physiol.

[68] You DM, Franzos MA, Holman RP (2008). Successful treatment of fulminant Clostridium difficile infection with fecal bacteriotherapy. Ann Intern Med.

[69] Aas J, Gessert CE, Bakken JS (2003). Recurrent Clostridium difficile colitis: case series involving 18 patients treated with donor stool administered via a nasogastric tube. Clin Infect Dis.

[70] Patel NC, Griesbach CL, DiBaise JK, Orenstein R (2013). Fecal microbiota transplant for recurrent Clostridium difficile infection: mayo clinic in Arizona experience. Mayo Clin Proc.

[71] Rohlke F, Surawicz CM, Stollman N (2010). Fecal flora reconstitution for recurrent Clostridium difficile infection: results and methodology. J Clin Gastroenterol.

[72] Persky SE, Brandt LJ (2000). Treatment of recurrent Clostridium difficile-associated diarrhea by administration of donated stool directly through a colonoscope. Am J Gastroenterol.

[73] Lawley TD, Clare S, Walker AW, Stares MD, Connor TR, Raisen C (2012). Targeted restoration of the intestinal microbiota with a simple, defined bacteriotherapy resolves relapsing *Clostridium difficile* disease in mice. PLoS Pathog.

[74] Buffie CG, Bucci V, Stein RR, McKenney PT, Ling L, Gobourne A (2014). Precision microbiome reconstitution restores bile acid mediated resistance to Clostridium difficile. Nature.

[75] Hamilton MJ, Weingarden AR, Sadowsky MJ, Khoruts A (2012). Standardized frozen preparation for transplantation of fecal microbiota for recurrent clostridium difficile infection. Am J Gastroenterol.

[76] Youngster I, Russell GH, Pindar C, Ziv-Baran T, Sauk J, Hohmann EL (2014). ORal, capsulized, frozen fecal microbiota transplantation for relapsing clostridium difficile infection. JAMA.

[77] Bakken JS (2014). Staggered and tapered antibiotic withdrawal with administration of kefir for recurrent clostridium difficile infection. Clin Infect Dis.

[78] Allen SJ, Wareham K, Wang D, Bradley C, Hutchings H, Harris W (2013). Lactobacilli and bifidobacteria in the prevention of antibiotic-associated diarrhoea and Clostridium difficile diarrhoea in older inpatients (PLACIDE): a randomised, double-blind, placebo-controlled, multicentre trial. Lancet.

[79] Kim K-H, Fekety R, Batts DH, Brown D, Cudmore M, Silva J (1981). Isolation of Clostridium difficile from the environment and contacts of patients with antibiotic-associated colitis. J Infect Dis.

[80] Lawley TD, Clare S, Walker AW, Goulding D, Stabler RA, Croucher N (2009). Antibiotic treatment of Clostridium difficile carrier mice triggers a supershedder state, spore-mediated transmission, and severe disease in immunocompromised hosts. Infect Immun.

[81] Cohen Stuart HMD, Gerding Dale NMD, Stuart Johnson MD, Kelly Ciaran PMD, Loo Vivian GMD, McDonald LCMD (2010). Clinical Practice Guidelines for Clostridium difficile Infection in Adults: 2010 Update by the Society for Healthcare Epidemiology of America (SHEA) and the Infectious Diseases Society of America (IDSA). Infect Cont Hosp Ep.

