# Supplementary material for: Effectiveness of fecal-derived microbiota transfer using orally administered capsules for recurrent Clostridium difficile infection
Source: BMC Infect Dis. 2015 Apr 17;15:191. doi: 10.1186/s12879-015-0930-z (PMC4506624; doi:10.1186/s12879-015-0930-z)
Supplement: Additional file 1: Table S2. — CDI history, risk factors, treatments and outcomes for patients receiving FMT. *This patient had continuous CDI diarrhea for one year despite antimicrobial treatment. **Initial improvement in symptoms was observed within 3 days; symptom improvement continued over the course of 2 weeks. [file 12879_2015_930_MOESM1_ESM.pdf]

Subject	CDI risk factors	Previous relapses	Previous CDI treatment	Previous hospital stays for CDI	Previous immuno-suppressive use	Previous PPI use	Previous history of FMT	Instance of treatment	Number of capsules	Dose (g input material)	Adverse effects observed	Time to symptom relief	Lasting cure
A	Ciprofloxacin for urinary tract infection; irritable bowel syndrome	3	vancomycin, fidaxomicin	No	No	No	No	Single treatment	9	1.1	Mild abdominal pain	2 days	Yes
B	Antibiotics for pyelonephritis/ urosepsis	4	vancomycin, metronidazole	No	No	No	Yes	Single treatment	15	3.6	None	3 days	Yes
C	Antibiotics for frequent respiratory infections	4	vancomycin	1	No	No	No	Single treatment	12	3.6	None	4 days	Yes
D	Antibiotics; mucositis	See legend*	vancomycin, IV tigeycline, IV metronidazole, fidaxomicin	1	Chemo-therapy	No	No	Single treatment	8	3.0	None	4 days	Yes
E	Antibiotics for diverticulitis	5	vancomycin, metronidazole, fidaxomicin	No	No	Yes	No	Single treatment	15	3.6	None	Gradual (see legend**)	Yes
F	Clindamycin for Group B streptococcus	4	metronidazole, vancomycin	1	No	No	No	Single treatment	12	3.6	None	Gradual	Yes
G	Antibiotics for respiratory tract infection	7	vancomycin, fidaxomicin	5	No	No	No	Single treatment	15	3.6	Moderate-to-severe abdominal pain	Gradual	Yes
H	Levofloxacin (multiple courses); previous norovirus enteritis; irritable bowel; ulcerative colitis; previous Asacol	4	vancomycin, metronidazole, fidaxomicin	No	No	No	No	Single treatment	8	3.6	None	Gradual	Yes
I	Antibiotics; prolonged healthcare facility stay	3	vancomycin (multiple courses), prolonged IV metronidazole	2	No	No	No	Single treatment	8	3.0	Mild abdominal pain	Gradual	Yes
J	Antibiotics; frequent hospitaliz-ation; chronic diarrhea; dehydration	3	vancomycin	No	No	Yes	Yes	Single treatment	10	1.1	None	Gradual	Yes
K	Antibiotics	2	vancomycin, metronidazole	No	No	No	No	Single treatment	6	1.1	None	Gradual	Yes
L	Antibiotics including ciprofloxacin for foot infection	3	vancomycin	3	No	Yes	No	Single treatment	8	1.1	None	Gradual	Yes
M	Antibiotics; prolonged healthcare facility stay	2	vancomycin	No	Methotrexate	Yes	No	Single treatment	10	8.3	None	Gradual	Yes
N	Ceftriaxone/azithromycin for respiratory infection	4	metronidazole, vancomycin	No	No	No	No	Single treatment	12	8.3	None	Gradual	Yes
O	Clindamycin for cellulitis	3	metronidazole, vancomycin, fidaxomicin	No	No	No	No	Single treatment	8	3.0	None	3 days	No
P	Intensive antibiotics for polymicrobial bacteremia	6	vancomycin, fidaxomicin	No	No	No	No	First	9	3.6	None	Gradual	No
								Second	10	8.3	None	Gradual	Yes
Q	Antibiotics; intestinal lymphoma	3	vancomycin, metronidazole, fidaxomicin	No	Chemo-therapy	No	No	First	10	1.1	None	Gradual	No
								Second	12	1.1	None	Gradual	Yes
R	Antibiotics	3	vancomycin, metronidazole, fidaxomicin	No	No	No	No	First	12	1.1	Mild abdominal pain	Gradual	No
								Second	10	1.1	None	Gradual	Yes
S	Antibiotics	>3	vancomycin, metronidazole, fidaxomicin, rifaximin	3	Chemo-therapy	No	No	First	6	1.1	Mild abdominal pain	3 days	No
								Second	8	1.1	Mild abdominal pain	None	No
								Third	10	1.1	None	None	No
								Fourth	22	3.6	Mild abdominal pain	None	No

Table 2. CDI history, risk factors, treatments and outcomes for patients receiving FMT.

*This patient had continuous CDI diarrhea for one year despite antimicrobial treatment.
**Initial improvement in symptoms was observed within 3 days; symptom improvement continued over the course of 2 weeks.
